# Management of choledocholithiasis with an ultraslim cholangioscope in a patient with possible anaphylaxis to contrast medium

**DOI:** 10.1055/a-2268-2470

**Published:** 2024-03-01

**Authors:** Kazuya Koizumi, Karen Kimura, Ryuhei Jinushi, Ryo Sato, Sakue Masuda

**Affiliations:** 1 Gastroenterology Medicine Center, Shonan Kamakura General Hospital, Kamakura, Japan

Adverse reactions to contrast medium during endoscopic retrograde cholangiopancreatography (ERCP) are rare 1 ; however, once they occur, the subsequent ERCP becomes challenging. Although alternatives using carbon dioxide or gadolinium exist 2 3 , resolution issues persist. Stone removal using a cholangioscope without fluoroscopy or contrast media in pregnant patients has been reported 4 ; however, due to the thickness and rigidity of conventional cholangioscopes, this remains problematic. We report on common bile duct (CBD) stone removal without contrast media, using a novel ultraslim cholangioscope with a tip diameter of 2.3 mm (DRES Slim Scope; Japan Lifeline Co., Ltd., Tokyo, Japan) ( Fig. 1 ) 5 .

**Fig. 1:**
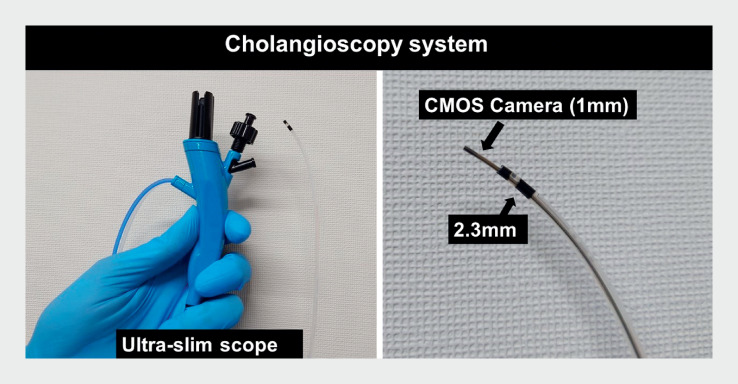
Cholangioscopy system. The cholangioscopy system involves inserting a camera (complementary metal oxide semiconductor type) with a diameter of 1 mm through an ultraslim scope with a diameter of 2.3 mm.

A 31-year-old woman presented to our department with recurrent abdominal pain and elevated biliary enzyme levels, suggesting choledocholithiasis. Computed tomography scans revealed no stones ( Fig. 2 ); however, endoscopic ultrasonography (EUS) performed under sedation after pre-administration of antibiotics detected CBD stones ( Fig. 3 ). With the patient’s condition stable post-EUS, we proceeded to ERCP.

**Fig. 2:**
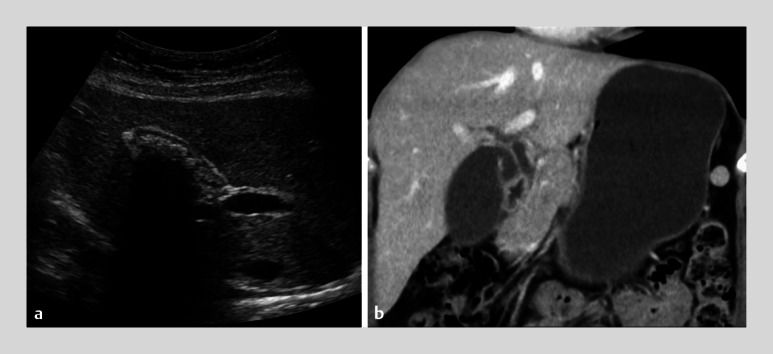
Imaging studies. **a** Ultrasonography showing gallbladder stones. **b** Computed tomography showing no biliary stones.

**Fig. 3:**
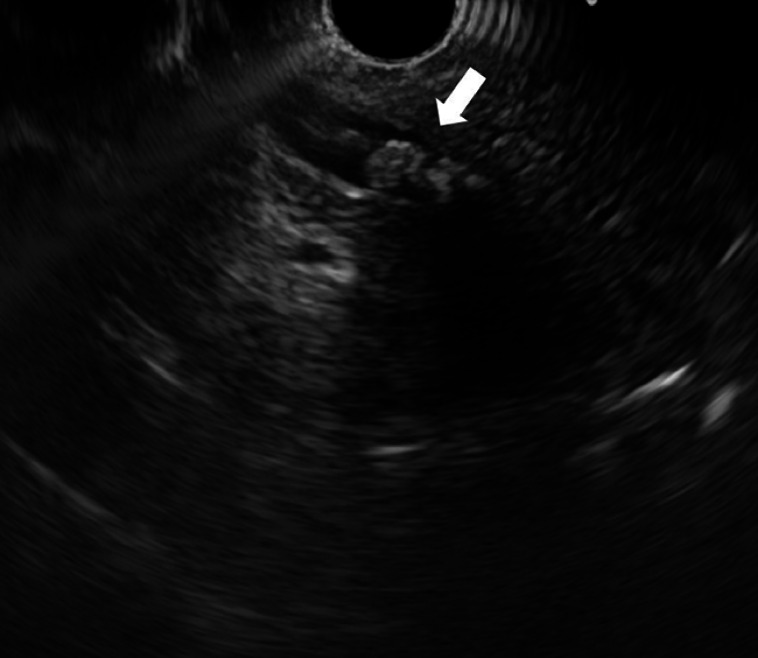
Endoscopic ultrasonography showing common bile duct stones.

Following contrast medium injection into the bile duct, a diagnosis of anaphylaxis was made due to lowering blood pressure, decreased oxygen saturation, and rash developing over the body. The procedure was stopped; epinephrine administration improved the patient’s condition. The contrast medium was likely responsible for the anaphylaxis, despite the possibility of effects from other medications.

On another day, a second ERCP was performed without contrast medium using an ultraslim cholangioscope. Initially, a guidewire was placed in the bile duct using an ultraslim cholangioscope, and then the cholangioscope was inserted. After confirming the presence of stones ( Fig. 4 **a** ), the cholangioscope was withdrawn and stones were removed in a standard manner using a basket catheter under fluoroscopic guidance without contrast medium or a cholangioscope ( Fig. 5 ). Finally, the ultraslim cholangioscope verified stone clearance ( Fig. 4 **b** , Video 1 ).

**Fig. 4:**
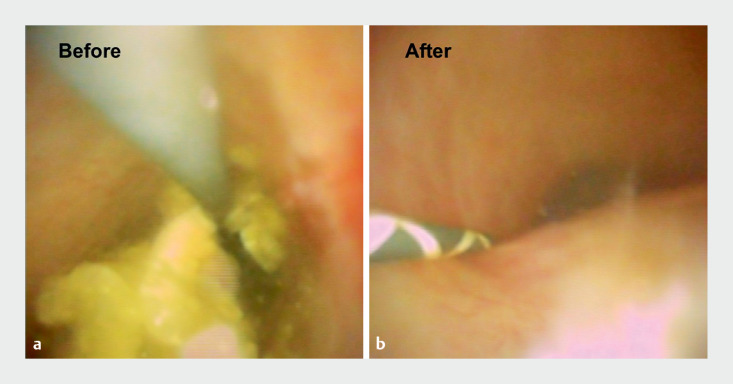
Cholangioscope images. **a** Stones were detected using an ultraslim scope. **b** After stone removal, the absence of stones in the bile duct was confirmed using the ultraslim scope.

**Fig. 5:**
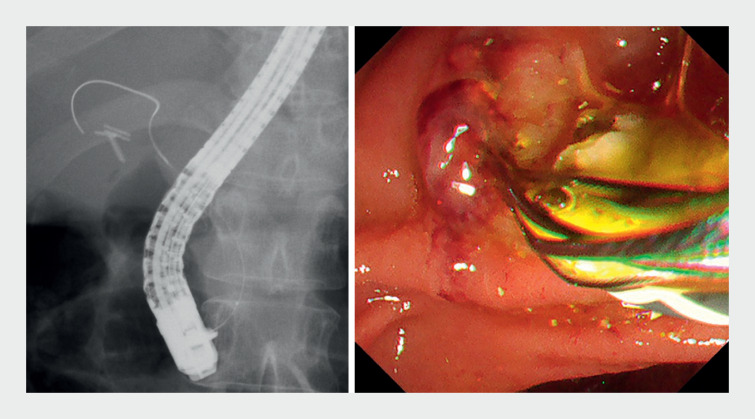
The stones were removed in a standard manner using a basket catheter under fluoroscopic guidance without contrast media or the cholangioscope.

Successful removal of common bile duct stones without contrast media using a novel ultraslim mother–baby cholangioscope.Video 1

Although ultraslim cholangioscopes do not allow stone removal under direct visualization because of the slim design, they are less invasive in confirming the absence of residual stones after stone removal.

Endoscopy_UCTN_Code_TTT_1AR_2AH

## Notes

Endoscopy E-Videos https://eref.thieme.de/e-videos
*E-Videos* is an open access online section of the journal *Endoscopy* , reporting on interesting cases and new techniques in gastroenterological endoscopy. All papers include a high-quality video and are published with a Creative Commons CC-BY license. Endoscopy E-Videos qualify for HINARI discounts and waivers and eligibility is automatically checked during the submission process. We grant 100% waivers to articles whose corresponding authors are based in Group A countries and 50% waivers to those who are based in Group B countries as classified by Research4Life (see: https://www.research4life.org/access/eligibility/ ). This section has its own submission website at https://mc.manuscriptcentral.com/e-videos .
